# Functional Neurological Disorder: Neurobiological Mechanisms, Biomarkers, and Integrated Treatment in a Female-Predominant Neuropsychiatric Condition

**DOI:** 10.3390/neurolint18060109

**Published:** 2026-06-02

**Authors:** Giuseppe Marano, Marianna Mazza

**Affiliations:** 1 Department of Neuroscience, Head-Neck and Chest, Section of Psychiatry, Fondazione Policlinico Universitario Agostino Gemelli IRCCS, Largo Agostino Gemelli 8, 00168 Rome, Italy; 2 Department of Neuroscience, Section of Psychiatry, Università Cattolica del Sacro Cuore, Largo Agostino Gemelli 8, 00168 Rome, Italy

**Keywords:** functional neurological disorder, predictive coding, sense of agency, brain network dysfunction, salience network, biomarkers, neuroimaging, neuropsychiatry, sex differences, multidisciplinary treatment

## Abstract

Background: Functional Neurological Disorder (FND) is a common and disabling condition at the interface of neurology and psychiatry, characterized by motor, sensory, seizure-like, or cognitive symptoms that are incongruent with recognized neurological disease but associated with substantial impairment. Despite its frequency and marked female predominance, FND remains underdiagnosed and often misunderstood. Methods: This narrative review synthesizes evidence from neurobiological, biomarker, and treatment studies, with attention to predictive coding, salience network dysfunction, impaired sense of agency, stress-related mechanisms, and sex- and gender-related vulnerability. Results: Current evidence supports a model of FND as a disorder of distributed brain network dysfunction involving abnormal interactions among salience, limbic, motor, and self-monitoring systems. Predictive coding and impaired agency models provide clinically useful frameworks for understanding symptom generation, although they remain mechanistic hypotheses rather than definitive causal explanations. Candidate biomarkers, including functional connectivity alterations, autonomic dysregulation, and HPA axis measures, offer pathophysiological insight but remain insufficiently validated for routine diagnosis. Female predominance likely reflects interacting biological, psychological, and sociocultural mechanisms rather than a single neuroendocrine pathway. Conclusions: This review contributes an integrated, clinically oriented framework linking neurobiology, biomarkers, sex/gender vulnerability, and treatment in FND. Current evidence supports multidisciplinary care combining diagnostic communication, specialized physiotherapy, psychotherapy, and coordinated follow-up, while future research should prioritize standardized phenotyping, longitudinal designs, and multimodal biomarker validation.

## 1. Introduction

Functional Neurological Disorder (FND) represents a common and disabling condition at the interface between neurology and psychiatry, accounting for a substantial proportion of outpatient neurological consultations [1,2]. Historically conceptualized as a “functional” or psychogenic disorder, FND has long been associated with diagnostic uncertainty, stigma, and fragmentation of care [3,4]. However, advances in cognitive neuroscience and neuroimaging have contributed to a transformative shift in perspective, supporting the view of FND as a disorder of brain network dysfunction involving altered self-agency, salience processing, and emotion–motor integration [5,6].

Notably, FND shows a marked female predominance, with women representing the majority of affected individuals across clinical and epidemiological studies [1,7]. Although this sex-related vulnerability has often been attributed to psychosocial factors, emerging evidence suggests a more complex interplay involving neurobiological, neuroendocrine, and stress-related mechanisms [8,9]. A better understanding of these mechanisms may provide important insights into the pathophysiology of FND and support the development of more personalized and integrated treatment approaches.

Despite increasing recognition of FND as a disorder of brain network dysfunction, several critical gaps remain in the current literature. In particular, the integration of neurobiological mechanisms with clinically applicable biomarkers is still limited, and no validated diagnostic or prognostic markers have yet been established [2,10]. Furthermore, although multiple models, ranging from predictive coding frameworks to stress-related neuroendocrine dysregulation, have been proposed, these approaches are often studied in isolation, with limited translation into unified and clinically actionable frameworks [3,9]. This theoretical fragmentation hinders the transition toward a precision medicine approach capable of accounting for the marked phenotypic heterogeneity of the disorder.

In addition, the marked female predominance of FND remains insufficiently explored from a mechanistic perspective. While epidemiological data consistently demonstrate higher prevalence rates in women, the contribution of sex-specific neurobiological factors, including hormonal modulation, stress responsivity, and differences in brain network organization, has not been systematically integrated into current models of the disorder [7,11]. Integrating these variables is essential not only for clarifying the etiology but also for optimizing clinical management strategies in a predominantly female population. Addressing this gap is therefore essential for advancing precision approaches to diagnosis and treatment.

In this context, the present narrative review aims to synthesize current evidence on the neurobiological mechanisms, emerging biomarkers, and integrated treatment strategies in FND, with a specific focus on its characterization as a female-predominant neuropsychiatric condition. By bridging advances in neuroscience, clinical research, and translational psychiatry, this work seeks to provide a comprehensive framework to inform both clinical practice and future research directions.

### Clinical Presentation and Phenotypes of Functional Neurological Disorder

FND encompasses a wide range of clinical presentations affecting motor, sensory, and cognitive domains. These symptoms are characterized by internal inconsistency and incongruence with recognized neurological diseases but are nonetheless associated with significant disability and distress [1,2]. Importantly, the diagnosis of FND is based on the identification of positive clinical signs rather than solely on the exclusion of structural pathology, representing a key shift in modern neurological practice [3]. This evidence-based approach allows for the bypass of the “diagnosis by exclusion” paradigm, thereby reducing diagnostic delays and improving patient acceptance of the condition.

Motor symptoms are among the most common manifestations and include functional weakness, tremor, dystonia, and gait disturbances. These presentations are often variable over time and influenced by attention or distraction, reflecting abnormal modulation of motor control [12]. For example, Hoover’s sign is a positive clinical sign used to identify functional limb weakness, based on the dissociation between voluntary and automatic motor activation [13].

Functional seizures, also referred to as psychogenic non-epileptic seizures (PNES), represent another frequent phenotype, characterized by paroxysmal events that resemble epileptic seizures but lack corresponding electrophysiological abnormalities [14].

Sensory symptoms, such as non-dermatomal sensory loss or visual disturbances, are also commonly reported and may reflect altered sensory processing and attentional mechanisms. In addition, functional speech and cognitive symptoms (including dysphonia, stuttering, and subjective cognitive impairment) highlight the involvement of higher-order networks related to language, attention, and self-referential processing [15]. The frequent coexistence of motor and nonmotor symptoms suggests a widespread vulnerability of the circuits regulating bodily awareness and executive control.

Despite their heterogeneity, these clinical manifestations share common underlying mechanisms involving abnormal interactions between motor, emotional, and cognitive systems. The main clinical presentations of FND, along with their characteristic features and key diagnostic signs, are summarized in Table 1.

## 2. Materials and Methods

This narrative review was conducted to synthesize current evidence on the neurobiological mechanisms, biomarkers, and treatment approaches in FND. A structured literature search was performed using major electronic databases, including PubMed, Scopus, and Web of Science.

The search strategy combined keywords related to FND (e.g., “functional neurological disorder”, “conversion disorder”, “psychogenic non-epileptic seizures”, “functional movement disorder”), neurobiology (e.g., “brain networks”, “predictive coding”, “self-agency”, “salience network”), biomarkers (e.g., “neuroimaging”, “functional connectivity”, “autonomic markers”, “HPA axis”, “cortisol”), treatment (e.g., “cognitive behavioral therapy”, “physiotherapy”, “multidisciplinary treatment”, “neuromodulation”), and etiological models (e.g., “biopsychosocial model”, “trauma”, “stress”, “sex differences”, “gender differences”). The inclusion of the term “biopsychosocial model” was intended to ensure that psychosocial, cultural, and gender-related determinants were considered alongside neurobiological mechanisms.

Studies published between January 2010 and March 2026 were considered, with priority given to recent literature published within the last 5–10 years to capture the latest advances in neuroimaging and translational neuroscience. Eligible studies included randomized controlled trials, systematic reviews, meta-analyses, and observational studies investigating neurobiological mechanisms, diagnostic markers, or therapeutic interventions in FND. Relevant experimental and translational studies were also included when they provided significant mechanistic insights.

Exclusion criteria comprised case reports, editorials, and studies not directly related to FND or its neurobiological and clinical characterization. Only articles published in English were considered. Although the present review was designed as a narrative rather than systematic review, a structured screening process was applied to improve transparency. Titles and abstracts retrieved from the database search were screened for relevance to the aims of the review. Full-text articles were then assessed when they addressed at least one of the following domains: clinical phenotypes of FND, neurobiological mechanisms, sex- or gender-related vulnerability, candidate biomarkers, or therapeutic interventions. Studies were prioritized when they provided original clinical data, neuroimaging or physiological findings, randomized or controlled intervention data, or high-quality systematic syntheses. No formal risk-of-bias scoring system was applied, because of the heterogeneity of study designs and the narrative scope of the review. However, greater interpretative weight was given to systematic reviews, meta-analyses, consensus recommendations, randomized or controlled trials, and studies with clearly defined diagnostic criteria.

Study selection was based on relevance to the aims of the review, with particular attention to studies addressing brain network dysfunction, sex-related differences, and clinically applicable biomarkers. The selection process prioritized studies with robust original data or those offering novel theoretical syntheses of existing neurobiological models.

Given the narrative nature of this review, no formal systematic review protocol, PRISMA flow diagram, meta-analysis, or quantitative risk-of-bias assessment was performed. This represents an intrinsic limitation of the present work. Nevertheless, the use of predefined search domains, explicit inclusion and exclusion criteria, and thematic synthesis was intended to improve methodological transparency while preserving the integrative scope required for a complex neuropsychiatric condition such as FND. Although this review does not follow a formal systematic review framework, efforts were made to ensure a comprehensive and balanced representation of the available evidence.

**Figure 1:**
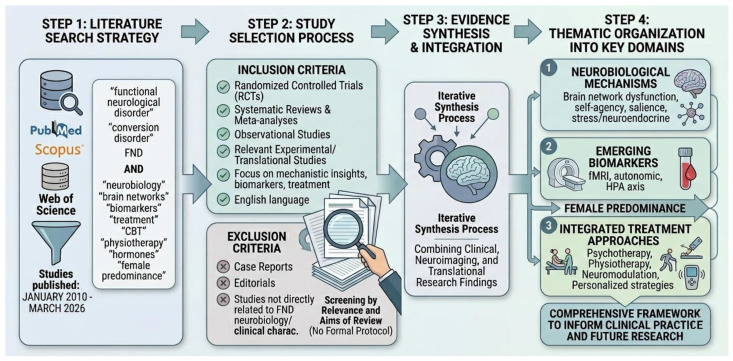
Conceptual framework of the narrative review methodology. Note. Schematic representation of the structured approach used for the selection and synthesis of scientific evidence in this review. Step 1 (Literature Search Strategy): Highlights the systematic search across PubMed, Scopus, and Web of Science using specific keywords related to Functional Neurological Disorder, neurobiology, and sex related differences for studies published from January 2010 to March 2026. Step 2 (Study Selection Process): Delineates the application of inclusion criteria such as randomized controlled trials and meta-analyses while excluding case reports or editorials to maintain a high standard of evidence. Step 3 (Evidence Synthesis and Integration): Represents the iterative process of combining findings from clinical research and neuroimaging to bridge the gap between bench and bedside. Step 4 (Thematic Organization into Key Domains): Outlines the final structure of the review which is divided into neurobiological mechanisms, emerging biomarkers, and integrated treatment approaches. This section emphasizes the overarching goal of providing a comprehensive framework to inform future research and clinical practice in the management of this neuropsychiatric condition.

The methodology of this narrative review follows a structured and multistage process designed to capture the complexity of Functional Neurological Disorder across different scientific disciplines. As illustrated in Figure 1, the conceptual framework begins with a comprehensive literature search across major electronic databases to identify high-quality evidence published between January 2010 and March 2026. This process involves the rigorous application of inclusion and exclusion criteria to ensure a focus on mechanistic insights and translational findings. The inclusion of the most recent data up to early 2026 ensures that the findings reflect the current state of the art in the field.

The subsequent iterative synthesis of the gathered evidence allows for the integration of clinical, neuroimaging, and neurobiological data into a unified perspective. This methodological pathway culminates in the thematic organization of the review into core domains encompassing neurobiological mechanisms, emerging biomarkers, and integrated therapeutic strategies with a specific emphasis on the female predominant nature of the condition. By adopting this multidimensional synthesis, the review aims to overcome the traditional silos of neurological and psychiatric research, fostering a more unified understanding of FND. All schematic figures included in this review should be interpreted as conceptual frameworks rather than validated causal maps. They synthesize current hypotheses and recurring findings from the literature, but the directionality and causal strength of several pathways remain incompletely established. Their purpose is to support clinical and theoretical integration, not to imply that all depicted mechanisms have been empirically confirmed in all FND phenotypes.

## 3. Epidemiology and Female Predominance

FND is a relatively common condition in neurological practice, accounting for approximately 5–10% of new outpatient neurology consultations and up to one-third of patients presenting with neurological symptoms not fully explained by structural disease [1,2]. The incidence of FND has been estimated at 4–12 cases per 100,000 persons per year, although variability across studies reflects differences in diagnostic criteria and clinical settings [8]. FND is associated with substantial disability, reduced quality of life, and high healthcare utilization, comparable to or exceeding that observed in many recognized neurological disorders [1]. The socioeconomic burden of FND is further compounded by the frequent delay in diagnosis, which often leads to unnecessary investigations and secondary iatrogenic complications.

A consistent and well-replicated finding across epidemiological studies is the marked female predominance of FND, with women representing approximately 60–75% of affected individuals [1,7]. This sex distribution appears stable across different clinical phenotypes, including functional movement disorders and psychogenic non-epileptic seizures, suggesting a shared underlying vulnerability rather than phenotype-specific effects [15]. Despite this robust observation, the mechanisms underlying female predominance remain incompletely understood.

Traditionally, this pattern has been attributed primarily to psychosocial factors, including higher exposure to trauma, stress, and adverse life events among women [8]. However, this explanation is likely insufficient to fully account for the observed sex differences. Emerging evidence points toward a more complex interplay involving biological sex, neuroendocrine regulation, and brain network organization [16]. For instance, sex-related differences in hypothalamic–pituitary–adrenal (HPA) axis responsivity, emotional processing, and salience attribution may contribute to increased vulnerability to functional symptoms [6,9]. Additional mechanisms, including the gut–brain axis, may further contribute to sex-related vulnerability, as the gut microbiota has been shown to modulate neuroendocrine, immune, and stress-related pathways in a sex-specific manner [17].

The interpretation of female predominance in FND requires caution. Current evidence does not support a single causal explanation, and direct studies testing sex-specific mechanisms in FND remain limited. Biological hypotheses involving hormonal modulation, HPA axis responsivity, stress sensitivity, immune-neuroendocrine interactions, and sex-related differences in salience or limbic network organization remain plausible but incompletely validated. Future studies should therefore distinguish clearly between biological sex-related mechanisms and gender-related sociocultural determinants, as these dimensions may interact but are not interchangeable.

A more comprehensive biopsychosocial model should also consider gender-related factors, including differential exposure to trauma and interpersonal violence, culturally shaped patterns of emotional expression, social expectations regarding bodily distress, and differences in help-seeking behavior. These variables may influence not only vulnerability to FND but also symptom reporting, referral pathways, diagnostic delay, and access to specialized care. Consequently, female predominance should be interpreted as the outcome of interacting biological, psychological, and sociocultural mechanisms rather than as evidence for a purely neuroendocrine or purely psychosocial model.

Neuroimaging studies suggest that women may exhibit distinct patterns of connectivity within limbic and salience networks, which are critically involved in emotion–motor integration and the sense of agency, key processes implicated in FND [15]. Hormonal factors, including fluctuations in estrogen and progesterone, have also been hypothesized to modulate stress reactivity and neural plasticity, although direct evidence in FND populations remains limited [18]. These hormonal influences are particularly relevant considering their capacity to alter GABAergic signaling and glutamatergic excitability within circuits governing motor control.

These lines of research support the view that female predominance in FND likely reflects a multifactorial vulnerability involving biological, psychological, and social determinants. A more systematic integration of sex-related factors into neurobiological models of FND may improve both diagnostic precision and the development of personalized therapeutic strategies. Moving beyond the traditional psychosocial narrative toward an integrated biological framework is a necessary step for destigmatizing the disorder and refining precision medicine protocols. Future research should include adequately powered cohorts stratified by sex and gender, longitudinal designs assessing hormonal status and life-stage factors, and standardized measures of trauma exposure, sociocultural context, psychiatric comorbidity, and healthcare access. Such studies would help clarify whether female predominance reflects differences in incidence, clinical expression, referral patterns, biological vulnerability, or a combination of these mechanisms.

## 4. Neurobiological Mechanisms of Functional Neurological Disorder

**Figure 2:**
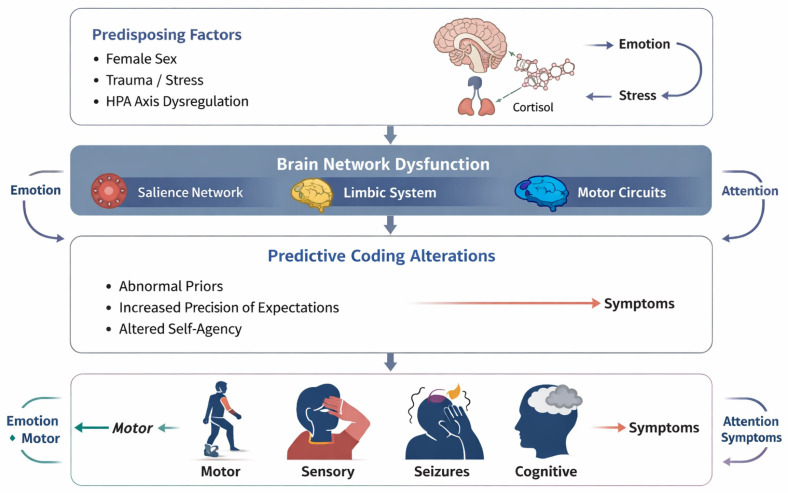
Integrated neurobiological model of functional neurological disorder. Note. The figure illustrates the interaction between predisposing factors (including sex-related vulnerability and stress mechanisms), brain network dysfunction, and predictive coding alterations leading to functional neurological symptoms. Dysfunction within salience, limbic, and motor networks contributes to abnormal self-agency and symptom perception. This model highlights the dynamic interplay between neurobiological and psychological processes in FND. This figure represents a conceptual synthesis of current evidence and hypotheses and should not be interpreted as a validated causal or diagnostic model.

The pathophysiology of FND is increasingly understood as a complex and dynamic process that transcends the traditional dichotomy between neurological and psychiatric domains. Rather than being localized to a single structural lesion, FND arises from the aberrant integration of sensory information, motor control, and emotional processing across distributed neural circuits. This modern neurobiological perspective emphasizes how systemic network dysfunction can manifest as objective neurological symptoms in the absence of classical structural damage. To provide a comprehensive overview of these interacting pathways, the integrated model of FND pathophysiology is illustrated in Figure 2. This framework encompasses the progression from underlying vulnerabilities to the active generation of symptoms through disrupted brain networks and altered cognitive processing. This transition from predisposition to clinical expression highlights the necessity of a multifaceted diagnostic approach.

### 4.1. Brain Network Dysfunction

FND is increasingly conceptualized as a disorder of brain network dysfunction rather than a structural neurological disease. Converging evidence from functional neuroimaging studies indicates abnormalities across multiple large-scale brain networks, particularly those involved in motor control, emotional regulation, and salience processing [6,15].

Key regions implicated include the supplementary motor area, anterior cingulate cortex, insula, amygdala, and prefrontal cortices, which together contribute to the integration of motor intentions, emotional states, and self-referential processing. Altered functional connectivity between limbic regions and motor circuits has been consistently reported, suggesting abnormal modulation of voluntary motor control by emotional and salience-related signals [9,19].

In particular, dysfunction within the salience network, comprising the anterior insula and anterior cingulate cortex, may play a central role in the aberrant prioritization of internal signals, leading to altered perception and amplification of bodily sensations [20]. This network-level disruption provides a neurobiological framework linking emotional processes to motor and sensory symptoms in FND. Furthermore, the failure of top-down inhibitory control over these networks likely facilitates the persistence of paroxysmal or continuous functional deficits.

### 4.2. Predictive Coding and Symptom Generation

One of the most influential theoretical models in FND is based on predictive coding, which conceptualizes the brain as a hierarchical inference system that continuously generates predictions about sensory inputs and updates them based on incoming information [3].

According to this framework, functional symptoms may arise from maladaptive prior expectations (“priors”) that are assigned excessive precision relative to sensory input. This imbalance leads to the dominance of internally generated predictions over external sensory evidence, resulting in the experience of symptoms that are subjectively real but not explained by structural pathology [21].

In FND, abnormal priors related to movement, sensation, or bodily states may be reinforced by attentional biases, prior experiences, or emotional salience. This may explain key clinical features such as symptom variability, suggestibility, and context-dependent expression [22]. Importantly, predictive coding models provide a mechanistic bridge between neurobiological processes and psychological factors, integrating cognitive, emotional, and perceptual dimensions of the disorder. This model effectively explains why directed attention toward a limb often exacerbates functional weakness, as it further increases the precision of the maladaptive prior. Although predictive coding provides a useful explanatory framework, it should not be interpreted as a definitive or exclusive model of FND. Existing evidence remains heterogeneous, and not all neuroimaging or experimental studies demonstrate uniform alterations in salience network activity, motor control circuits, or agency-related regions. Differences in clinical phenotype, symptom duration, psychiatric comorbidity, trauma exposure, medication status, task design, and analytic methodology may partly explain inconsistent findings across studies. Moreover, predictive coding models remain difficult to test directly in routine clinical settings, because constructs such as “abnormal priors” and “precision weighting” are inferential and cannot yet be measured through a single validated clinical biomarker. Therefore, these models are best understood as mechanistic hypotheses that integrate clinical observations with network neuroscience, rather than as established causal explanations for all FND presentations.

### 4.3. Impaired Sense of Agency

A central feature of FND is the disruption of the sense of agency, the subjective experience of initiating and controlling one’s own actions. Neurobiological models suggest that this impairment reflects altered integration between motor intention signals and sensory feedback, mediated by distributed cortical networks [6].

Functional neuroimaging studies have demonstrated abnormal activation patterns in regions associated with self-monitoring and motor awareness, including the temporoparietal junction, supplementary motor area, and prefrontal cortex. These alterations may lead to a mismatch between predicted and actual sensory consequences of actions, contributing to the experience of involuntary movements or loss of control over bodily functions [23]. It is important to stress that impaired agency in FND is not equivalent to malingering or voluntary control but rather reflects a neurobiologically grounded disturbance in self-referential processing. This distinction is critical for both clinical understanding and patient communication. The objective identification of this mismatch via neuroimaging provides a powerful tool for validating the patient’s lived experience of involuntariness. From a clinical perspective, impaired sense of agency can be understood as a mismatch between the patient’s intention to move, the predicted sensory consequences of that movement, and the actual sensory feedback generated by the body. In healthy voluntary movement, motor intention is accompanied by an internal prediction that allows the action to be experienced as self-generated. In FND, this predictive process may become disrupted: movement may occur without being experienced as fully voluntary, or conversely, an intended movement may fail to be executed because abnormal expectations and excessive self-focused attention interfere with automatic motor control. This framework helps explain why symptoms such as functional tremor, weakness, or gait disturbance may worsen under direct attention and improve with distraction. Importantly, this mechanism does not imply conscious production of symptoms, but rather a disturbance in the neural processes that normally link intention, movement, sensory feedback, and self-attribution.

### 4.4. Emotion–Motor Interaction and Stress Mechanisms

Emotional processing and stress-related mechanisms play a crucial role in the pathophysiology of FND. Increased connectivity between limbic regions, such as the amygdala, and motor control areas suggests that emotional salience may directly influence motor output [9,24].

Dysregulation of the HPA axis has also been implicated, with evidence of altered stress responsivity and abnormal cortisol patterns in patients with FND [8]. Chronic stress and trauma may contribute to sensitization of neural circuits involved in threat detection and bodily awareness, reinforcing maladaptive predictions and symptom persistence [25].

These findings are consistent with accumulating evidence supporting a bidirectional relationship between psychiatric disorders and systemic physiological processes, particularly cardiovascular function, as conceptualized within the brain–heart axis framework [26].

It is therefore possible to imagine a model in which emotional, cognitive, and motor systems interact dynamically, leading to the emergence and maintenance of functional symptoms. Rather than representing purely psychological or neurological phenomena, FND symptoms arise from the integration of these processes within distributed brain networks. The chronic activation of stress pathways likely lowers the threshold for network instability, allowing minor physical or emotional triggers to precipitate full symptomatic episodes.

**Figure 3:**
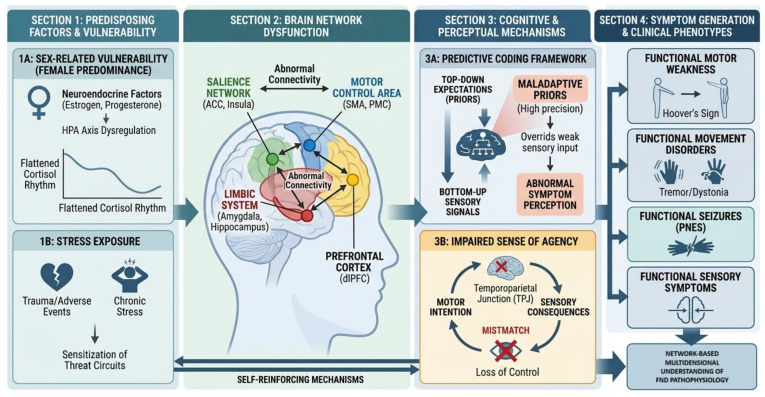
Integrated neurobiological model of functional neurological disorder. Note. Comprehensive schematic representation of the hierarchical and interacting mechanisms driving the pathophysiology of Functional Neurological Disorder. Section 1 (Predisposing Factors and Vulnerability): Highlights the foundational role of biological sex and neuroendocrine factors alongside psychosocial stressors. This section emphasizes how a female predominant vulnerability and HPA axis dysregulation create a biological soil for the development of the disorder. Section 2 (Brain Network Dysfunction): Delineates the core circuits involved in symptom expression including the salience network, the limbic system, and motor control areas. It illustrates the abnormal connectivity between the amygdala and the supplementary motor area which facilitates the emotional modulation of motor output. Section 3 (Cognitive and Perceptual Mechanisms): Focuses on the predictive coding framework where top-down expectations or priors dominate bottom-up sensory signals. This section also depicts the impaired sense of agency resulting from a mismatch between motor intentions and sensory feedback mediated by the temporoparietal junction. Section 4 (Symptom Generation and Clinical Phenotypes): Represents the final output of these integrated dysfunctions leading to the diverse clinical manifestations of the disorder. The bidirectional arrows indicate the self-reinforcing nature of these mechanisms where symptom perception further strengthens maladaptive neural priors and stress responses. Abbreviations: HPA axis, Hypothalamic–Pituitary–Adrenal axis; ACC, Anterior Cingulate Cortex; SMA, Supplementary Motor Area; PMC, Premotor Cortex; dlPFC, dorsolateral Prefrontal Cortex. This figure represents a conceptual synthesis of current evidence and hypotheses and should not be interpreted as a validated causal or diagnostic model.

The convergence of these diverse pathways suggests that FND is not the result of a single localized deficit but rather emerges from a complex and dynamic interaction across multiple biological levels. As synthesized in Figure 3, this integrated neurobiological model illustrates how predisposing factors such as sex related vulnerability and chronic stress exposure set the stage for large-scale network dysfunction. Within this framework, the synergy between limbic hyperactivation and impaired salience processing disrupts the standard mechanisms of motor control and self-awareness. This disruption is further compounded by maladaptive predictive coding, where excessive precision is assigned to internal priors over actual sensory evidence. By bridging the gap between emotional processing and the physical manifestation of symptoms, this multidimensional perspective provides a robust foundation for understanding the heterogeneous clinical presentations of the disorder. This systemic integration represents the current gold standard for conceptualizing FND in both research and clinical settings.

## 5. Biomarkers in Functional Neurological Disorder

The identification of reliable biomarkers in FND represents a critical challenge for transitioning from group-level observations to individualized clinical care. As FND is increasingly recognized as a network-level disturbance, the search for biomarkers has expanded beyond traditional structural imaging to encompass dynamic measures of brain function, autonomic reactivity, and neuroendocrine signaling.

**Figure 4:**
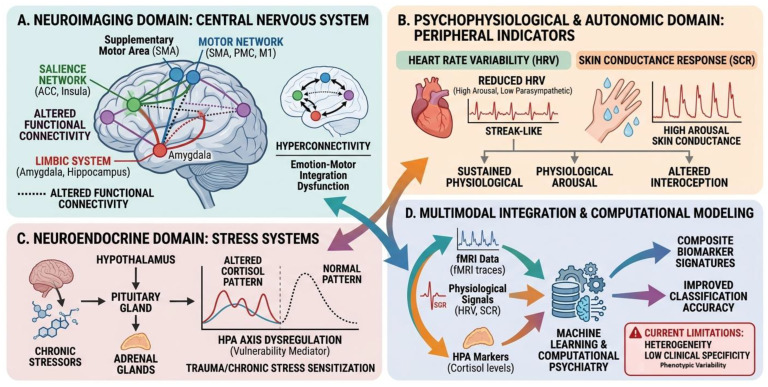
Multimodal biomarker framework in Functional Neurological Disorder. Note. Schematic representation of the main biomarker domains investigated in Functional Neurological Disorder and their integration into a unified research framework. Neuroimaging Domain illustrates key neural signatures including altered functional connectivity within the salience, limbic, and motor networks, highlighting nodes such as the amygdala and supplementary motor area. Psychophysiological and Autonomic Domain outlines peripheral indicators of nervous system dysregulation such as heart rate variability and skin conductance which reflect sustained physiological arousal. Neuroendocrine domain delineates the role of hypothalamic–pituitary–adrenal axis markers, focusing on altered cortisol patterns as biological mediators of vulnerability. Multimodal Integration represents the emerging shift toward combining these distinct data types through computational modeling to improve classification accuracy despite current limitations in clinical specificity. Abbreviations. ACC, anterior cingulate cortex; SMA, supplementary motor area; PMC, premotor cortex; M1, primary motor cortex; HPA axis, hypothalamic–pituitary–adrenal axis; fMRI, functional magnetic resonance imaging. This figure represents a conceptual synthesis of current evidence and hypotheses and should not be interpreted as a validated causal or diagnostic model.

These markers aim not only to support the positive diagnosis of FND but also to provide objective measures of disease severity, treatment response, and underlying biological vulnerability. Although no single gold standard marker has yet been validated for routine clinical use, the integration of multiple biological signals offers a promising pathway toward a more objective and mechanistic understanding of the disorder. This shift toward objective quantification is essential for moving the field beyond subjective symptom reporting and toward a biologically grounded diagnostic framework. To summarize the multidimensional nature of biomarker research in Functional Neurological Disorder, including neuroimaging, autonomic, and neuroendocrine domains, an integrated overview is presented in Figure 4.

### 5.1. Neuroimaging Biomarkers

Neuroimaging represents the most extensively studied domain for biomarker discovery in Functional Neurological Disorder. Functional magnetic resonance imaging (fMRI) studies have consistently identified alterations in functional connectivity across brain networks implicated in emotion regulation, motor control, and self-referential processing [6,15]. Abnormal interactions between limbic regions (e.g., amygdala, insula) and motor areas (e.g., supplementary motor area) have been proposed as a potential neural signature of FND, reflecting the influence of emotional salience on motor output. Altered activity within the salience network and default mode network has also been reported, suggesting disruptions in attention, internal monitoring, and bodily awareness [5,27]. These findings suggest that FND may be characterized by a failure of the prefrontal cortex to adequately regulate limbic motor crosstalk, particularly during high arousal states.

Despite these findings, neuroimaging biomarkers in FND remain largely at the group level and are not yet suitable for individual diagnosis. Variability across study designs, small sample sizes, and heterogeneity of clinical phenotypes limit their clinical applicability. As such, neuroimaging should currently be considered a promising research tool rather than a validated diagnostic marker.

### 5.2. Psychophysiological and Autonomic Biomarkers

Increasing attention has been directed toward psychophysiological and autonomic markers, particularly those reflecting stress responsivity and arousal regulation. Patients with FND have been shown to exhibit alterations in autonomic nervous system activity, including changes in heart rate variability, skin conductance, and startle responses [10].

These findings are consistent with models implicating heightened sensitivity to internal bodily signals and dysregulated interoception. Abnormal autonomic patterns may reflect a state of sustained physiological arousal, which could contribute to symptom generation and maintenance [28,29]. The presence of autonomic hyperarousal even in the absence of conscious emotional distress supports the hypothesis that FND involves a physiological decoupling between subjective experience and bodily states.

Similar to neuroimaging findings, these markers lack specificity and are shared with other psychiatric and neurological conditions. Their role may therefore be more relevant for understanding pathophysiology and monitoring treatment response rather than establishing a definitive diagnosis.

### 5.3. Neuroendocrine Biomarkers and Stress Systems

Dysregulation of the HPA axis has been proposed as a key neurobiological mechanism underlying vulnerability to FND. Altered cortisol secretion patterns, including both hyper- and hypo-reactivity, have been observed in patients with functional symptoms, particularly in the context of chronic stress or trauma exposure [30].

These neuroendocrine alterations may influence brain networks involved in emotion regulation and salience attribution, thereby reinforcing maladaptive predictive processes [31]. Moreover, sex-related differences in HPA axis function may partially contribute to the observed female predominance of FND, although direct evidence remains limited. The modulation of neural plasticity by chronic corticosteroid exposure may create a permissive environment for the consolidation of abnormal motor or sensory priors.

Overall, neuroendocrine markers provide a biologically plausible link between stress, brain function, and symptom expression, but their clinical utility as standalone biomarkers remains uncertain.

### 5.4. Toward Integrated and Multimodal Biomarkers

Given the complexity and heterogeneity of FND, it is unlikely that a single biomarker will be sufficient for diagnostic or prognostic purposes. Instead, current research is increasingly focused on multimodal approaches that integrate neuroimaging, physiological, and behavioral data [32].

Advances in computational psychiatry and machine learning may facilitate the identification of composite biomarker signatures capable of capturing individual variability and improving classification accuracy. However, most existing studies remain exploratory, and there is a need for large-scale, longitudinal, and clinically validated research to translate these approaches into routine practice. The future of biomarker discovery in FND likely lies in the development of systemic algorithms that can account for the dynamic interactions between central neural networks and peripheral physiological signals.

At present, biomarkers in FND should be interpreted as tools for advancing mechanistic understanding and supporting personalized care, rather than as definitive diagnostic criteria. Current research on biomarkers in FND spans multiple domains, including neuroimaging, autonomic physiology, and neuroendocrine systems. However, most findings remain exploratory and lack sufficient validation for clinical use.

Several factors currently limit the reproducibility and translational value of biomarker studies in FND. FND is clinically heterogeneous, and studies often combine patients with functional seizures, motor symptoms, sensory symptoms, and mixed phenotypes, despite likely differences in underlying mechanisms. Sample sizes are frequently small, reducing statistical power and increasing the risk of overestimating effect sizes. Neuroimaging studies vary substantially in acquisition protocols, task paradigms, preprocessing pipelines, and analytic strategies, making cross-study comparison difficult. In addition, psychiatric comorbidities, trauma history, medication exposure, symptom chronicity, and illness beliefs are not always consistently measured or controlled. Finally, most biomarkers have been identified at the group level and have not yet demonstrated sufficient sensitivity, specificity, or predictive value for individual diagnosis or prognosis. Future research should therefore prioritize larger multicenter cohorts, standardized phenotyping, preregistered analytic pipelines, longitudinal follow-up, and validation of biomarker models in independent samples.

A summary of the main biomarker domains, their potential applications, and current limitations is provided in Table 2.

## 6. Treatment Approaches in Functional Neurological Disorder

**Figure 5:**
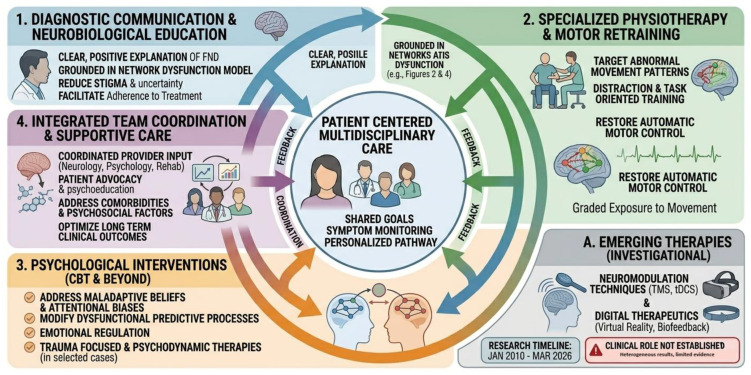
Integrated multidisciplinary treatment model for Functional Neurological Disorder. Note. Schematic overview illustrating the coordinated and multifaceted therapeutic approach for Functional Neurological Disorder. Diagnostic Communication and Psychoeducation represents the foundational step of treatment. It emphasizes providing patients with a clear, non-stigmatizing explanation of FND grounded in neurobiological concepts like brain network dysfunction to foster therapeutic engagement. Specialized Physiotherapy and Motor Retraining delineates interventions for functional motor symptoms. This domain focuses on restoring automatic movement patterns through distraction techniques, graded exposure, and task-oriented training, explicitly targeting abnormal control mechanisms. Psychological Interventions (CBT and Beyond) outlines evidence-based approaches such as Cognitive Behavioral Therapy (CBT) to address maladaptive beliefs and attentional biases. It also includes trauma-focused and psychodynamic therapies for patients where emotional dysregulation or adverse life events are prominent. Multidisciplinary Coordination and Personalized Care illustrates the central integration of neurological, psychological, and rehabilitation provider input. This section highlights the importance of tailored treatment plans that account for individual cognitive, emotional, and social factors to optimize long-term clinical outcomes. Emerging Therapies and Neuromodulation acknowledges investigational tools, including transcranial magnetic stimulation and digital therapeutics like virtual reality, which aim to directly modulate dysfunctional neural circuits and enhance engagement in retraining protocols. This figure represents a conceptual synthesis of current evidence and hypotheses and should not be interpreted as a validated causal or diagnostic model.

The management of FND has undergone a significant paradigm shift, moving away from purely symptomatic or exclusionary approaches towards integrated, neurobiologically informed treatment models. Modern therapeutic strategies recognize FND as a consolidated neuropsychiatric condition that requires a multidisciplinary framework to address its complex physiological and psychological mechanisms. This comprehensive approach emphasizes the importance of personalized care pathways, beginning with effective diagnostic communication and extending to specialized physical and psychological therapies aimed at retraining aberrant brain networks and restoring functional independence. This shift reflects a deeper understanding of the brain as a plastic organ where functional circuits can be reorganized through targeted intervention. To provide a comprehensive overview of current therapeutic strategies and their integration within multidisciplinary care models, the main treatment domains in Functional Neurological Disorder are illustrated in Figure 5.

### 6.1. Principles of Treatment

The management of FND requires a multidisciplinary and individualized approach, reflecting its complex neurobiological and psychosocial underpinnings. Current evidence supports a shift from purely symptomatic management toward integrated models that address both neural mechanisms and functional impairment [2,33,34].

A fundamental component of treatment is effective communication of the diagnosis, which has been shown to improve patient understanding, engagement, and clinical outcomes. Providing a clear, non-stigmatizing explanation grounded in neurobiological models, such as brain network dysfunction, can help reduce uncertainty and facilitate adherence to treatment [1,35].

### 6.2. Cognitive Behavioral Therapy and Psychotherapeutic Interventions

Cognitive Behavioral Therapy (CBT) represents one of the most studied and evidence-based psychotherapeutic approaches for FND. CBT interventions are typically tailored to address maladaptive beliefs, attentional biases, and symptom-focused behaviors, with the aim of modifying dysfunctional predictive processes and improving emotional regulation [36].

In patients with functional seizures and other FND presentations, CBT has been associated with reductions in symptom frequency, improvements in quality of life, and decreased psychological distress. However, treatment response is variable, and outcomes may depend on factors such as symptom chronicity, comorbid psychiatric conditions, and patient engagement [37]. Addressing the patient’s metacognitive awareness of symptom triggers is often a core element of successful CBT protocols.

Other psychotherapeutic approaches, including trauma-focused therapies and psychodynamic interventions, may be beneficial in selected patients, particularly when adverse life events or emotional dysregulation play a prominent role [38]. These approaches focus on unconscious processes, emotional conflicts, and maladaptive patterns of affect regulation that may contribute to symptom formation [39,40]. Contemporary psychodynamic models emphasize the role of impaired emotional awareness, alexithymia, and difficulties in symbolization, which may lead to the expression of psychological distress through bodily symptoms. In this framework, functional symptoms can be understood as meaningful, though maladaptive, expressions of internal conflict [41]. The empirical evidence supporting psychodynamic interventions in FND remains limited compared to cognitive behavioral therapy, with most studies consisting of small samples or observational designs. As such, these approaches may be considered in selected patients, particularly when emotional processing difficulties or trauma-related factors are prominent, but further research is needed to establish their efficacy within standardized treatment pathways. Nevertheless, these therapies provide a critical depth of treatment for patients whose network dysfunction is deeply rooted in early relational trauma.

### 6.3. Physiotherapy and Motor Retraining

Physiotherapy is a cornerstone of treatment, particularly for patients with functional motor symptoms. Modern physiotherapeutic approaches focus on retraining movement patterns, restoring automatic motor control, and reducing maladaptive attention to symptoms. Techniques often include distraction, task-oriented training, and graded exposure to movement, aiming to normalize motor output and improve functional independence. Evidence suggests that specialized physiotherapy programs can lead to significant improvements in motor function and disability, especially when delivered within a multidisciplinary framework [42,43]. Importantly, physiotherapy in FND differs from standard neurological rehabilitation, as it explicitly targets abnormal movement patterns rather than structural deficits.

### 6.4. Multidisciplinary and Integrated Care Models

Increasing evidence supports the effectiveness of multidisciplinary treatment models that integrate neurological, psychological, and rehabilitation approaches. These models emphasize coordinated care, patient-centered planning, and continuity across clinical settings [2,42,44].

Integrated interventions combining CBT, physiotherapy, and psychoeducation have demonstrated improvements in both symptom severity and functional outcomes, highlighting the importance of addressing multiple domains simultaneously. Such approaches are particularly relevant given the heterogeneity of FND and the need to tailor treatment to individual profiles, including cognitive, emotional, and social factors.

In practical terms, an integrated care pathway for FND should begin with a confident positive diagnosis based on recognized clinical signs, followed by clear communication that validates symptom reality while explaining the disorder in terms of reversible brain network dysfunction. Early referral to clinicians familiar with FND is important to avoid repeated investigations and contradictory explanations. Treatment planning should then be individualized according to the dominant phenotype: patients with motor symptoms may benefit primarily from specialized physiotherapy and motor retraining, whereas patients with functional seizures may require seizure-specific CBT and psychoeducation. Comorbid depression, anxiety, trauma-related symptoms, pain, fatigue, and sleep disturbance should be actively screened and treated in parallel.

A coordinated multidisciplinary model should include regular communication between neurologists, psychiatrists or psychologists, physiotherapists, occupational therapists, and primary care providers. Shared treatment goals should be functional and measurable, such as improving walking distance, reducing emergency visits, increasing activity tolerance, or decreasing seizure frequency. Follow-up should monitor both symptom severity and functional recovery, avoiding excessive reinforcement of symptom-focused attention. This stepped-care approach may allow patients with milder or recent-onset symptoms to receive focused education and targeted rehabilitation, while those with chronic, severe, or comorbid presentations can be referred to specialized multidisciplinary programs.

### 6.5. Emerging Therapies and Neuromodulation

Emerging therapeutic approaches include neuromodulation techniques such as transcranial magnetic stimulation (TMS) and transcranial direct current stimulation (tDCS), which aim to modulate dysfunctional brain networks involved in motor control and emotional regulation [45,46].

Preliminary studies suggest potential benefits, particularly in motor symptoms, but evidence remains limited and heterogeneous. Standardization of protocols and larger randomized controlled trials are required before these techniques can be routinely recommended. Neuromodulation remains investigational, and its clinical role is not yet established.

Digital and technology-assisted interventions, including virtual reality and biofeedback, are also being explored as adjunctive tools to enhance engagement and facilitate motor and cognitive retraining. However, their role in clinical practice is still evolving. These innovations may eventually provide a way to target the specific neural nodes of the salience network identified in neuroimaging studies.

## 7. Discussion

FND is increasingly recognized as a condition arising from the interaction of neurobiological, psychological, and social factors, rather than a purely psychogenic or purely neurological entity [2,3]. The evidence reviewed in this paper supports a model of FND as a disorder of brain network dysfunction, in which altered predictive processes, impaired sense of agency, and abnormal salience attribution contribute to symptom generation and persistence [6,15]. This paradigm shift acknowledges that while the structural integrity of the brain may be preserved, the dynamic communication between its nodes is fundamentally compromised.

A key implication of this framework is the need to move beyond traditional dichotomies between “organic” and “functional” disorders. Instead, FND may be better understood within a dimensional and network-based perspective, in which symptoms emerge from dysregulated interactions across distributed neural systems [3]. This conceptual shift has important clinical consequences, particularly in reducing stigma and improving diagnostic communication [1]. By validating the biological reality of functional symptoms, clinicians can foster a more collaborative therapeutic alliance, which is often the first step toward recovery.

Despite growing advances in neuroimaging and computational models, the identification of reliable biomarkers remains an unresolved challenge. Current findings, ranging from altered functional connectivity to autonomic and neuroendocrine dysregulation, provide important insights into pathophysiology but lack sufficient specificity and reproducibility for routine clinical use [10]. The heterogeneity of FND presentations further complicates biomarker discovery, highlighting the need for multimodal and longitudinal approaches. Specifically, the transition from group-level statistics to individual predictive signatures represents the next frontier in FND research.

The marked female predominance observed in FND represents another critical but underexplored dimension. While traditionally attributed to psychosocial vulnerability, emerging evidence suggests that sex-related differences in stress responsivity, hormonal modulation, and brain network organization may play a significant role [8,9]. However, current data remain limited, and future research should aim to systematically integrate sex and gender variables into neurobiological models and clinical trials. Investigating the neuroprotective or sensitizing effects of sex hormones on neural plasticity may clarify why women are disproportionately represented in clinical cohorts. In addition to biological and neuroendocrine mechanisms, sociocultural factors are important for understanding FND vulnerability, clinical presentation, and care pathways. Cultural narratives about illness, gender roles, stigma surrounding psychiatric symptoms, and differences in access to neurological and psychological care may influence how symptoms are experienced, reported, and interpreted by clinicians. These factors may also contribute to diagnostic delay and to variability in treatment engagement. A comprehensive biopsychosocial model of FND should therefore integrate neurobiological vulnerability with developmental history, trauma exposure, interpersonal context, cultural meaning, and healthcare system factors.

From a therapeutic perspective, the evidence supports the use of integrated, multidisciplinary approaches combining cognitive behavioral therapy, physiotherapy, and patient-centered neurological care [2,12]. Emerging treatments, including neuromodulation, offer promising avenues but require further validation in large-scale studies [45,47]. The success of these interventions likely depends on their ability to target the specific network nodes responsible for the patient’s individual phenotype.

Importantly, several limitations should be acknowledged. The majority of available studies are characterized by small sample sizes, heterogeneity in diagnostic criteria, and variability in outcome measures [10]. Moreover, many findings remain at the group level and lack direct applicability to individual patients.

Future directions should focus on the development of integrated models combining neurobiological, behavioral, and clinical data, with particular attention to personalized approaches to diagnosis and treatment. Advances in computational psychiatry and multimodal data integration may contribute to identifying clinically meaningful subtypes of FND [2].

**Figure 6:**
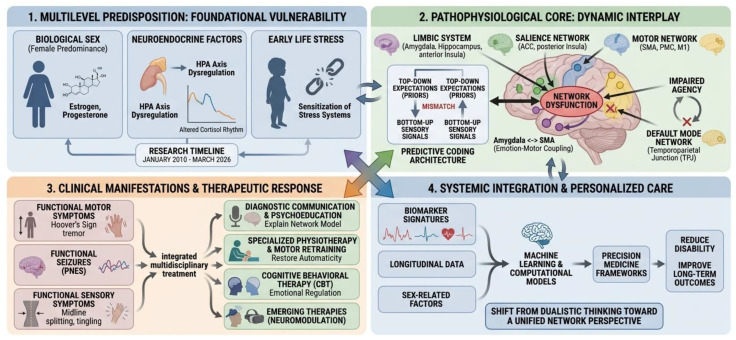
Global integrative framework of Functional Neurological Disorder. Note. A comprehensive synthesis of the biopsychosocial and network-based dimensions of FND. Multilevel Predisposition illustrates the intersection of biological sex, neuroendocrine factors such as the HPA axis, and early life stress as the foundational vulnerability. Pathophysiological Core highlights the dynamic interplay between brain network dysfunction including Salience, Limbic, and Motor circuits and the cognitive architecture of symptom generation through Predictive Coding and Agency. Clinical and Therapeutic Convergence maps the transition from diverse clinical phenotypes to an integrated multidisciplinary treatment response, emphasizing the bidirectional flow between neurobiological retraining and psychological regulation. Systemic Integration: the model advocates for a shift from dualistic thinking toward a unified network perspective that informs personalized clinical practice. Abbreviations. HPA axis, hypothalamic-pituitary-adrenal axis. This figure represents a conceptual synthesis of current evidence and hypotheses and should not be interpreted as a validated causal or diagnostic model.

In order to encapsulate the multifaceted evidence discussed throughout this review, Figure 6 presents a unified conceptual framework. This model serves to synthesize the intricate convergence of neurobiological networks, psychological processes, and clinical interventions, providing an integrated and systemic map of the current state of Functional Neurological Disorder as a paradigmatic neuropsychiatric entity.

## 8. Conclusions

The contemporary understanding of FND has undergone a profound transformation, moving away from exclusionary labels toward a positive and mechanism-based definition. FND should be formally conceptualized as a complex neuropsychiatric condition characterized by the dysfunction of distributed brain networks rather than as a purely functional or psychogenic disorder lacking a biological foundation. While remarkable advances in cognitive neuroscience and functional neuroimaging have improved our understanding of its underlying mechanisms specifically regarding the roles of maladaptive predictive coding, impaired sense of agency, and limbic motor hyperconnectivity significant challenges remain in translating these insights into validated biomarkers and standardized treatments that can be applied with precision to the individual patient.

The evidence presented throughout this review underscores that a personalized and integrated approach, combining transparent neurological diagnostic communication, evidence-based psychological interventions, and specialized rehabilitative strategies, represents the most effective current model of care. Furthermore, the striking female predominance in FND highlights a critical need for future research to move beyond psychosocial explanations and prioritize the systematic investigation of sex related biological variables, including neuroendocrine modulation and sex specific brain network organization. Future research should therefore prioritize the development of multimodal biomarker signatures, the execution of longitudinal studies to track treatment response, and the integration of these factors to advance precision medicine frameworks that can ultimately reduce disability and improve the long-term functional outcomes for all patients affected by FND. Only through such a systemic and rigorous approach can the clinical community offer truly transformative care for this historically underserved population.

## Figures and Tables

**Table 1:** Main Clinical Presentations of Functional Neurological Disorder.

Clinical Presentation	Typical Features	Key Positive Signs	Neurobiological Correlates (Hypothesized)	Selected References
Functional motor weakness	Sudden or fluctuating weakness, often inconsistent with neuroanatomy	Hoover’s sign, give-way weakness	Altered motor network connectivity; impaired voluntary motor control	[12,13,15]
Functional movement disorders (tremor, dystonia, gait)	Variable, distractible, incongruent movements	Entrainment, variability, distractibility	Abnormal emotion–motor interaction; salience network dysfunction	[12,15]
Functional seizures (PNES)	Episodes resembling epileptic seizures without EEG correlates	Closed eyes, asynchronous movements, prolonged duration	Limbic hyperactivation; altered stress response	[14,15]
Functional sensory symptoms	Non-dermatomal sensory loss, visual or auditory disturbances	Midline splitting, inconsistency on testing	Altered sensory prediction and attention	[13,15]
Functional speech disorders	Dysphonia, stuttering, or aphonia without structural cause	Inconsistency, task-dependent changes	Disrupted motor–language integration	[15]
Functional cognitive symptoms (“brain fog”)	Attention, memory, and executive complaints	Discrepancy between subjective and objective findings	Altered network connectivity; attentional dysregulation	[15]

**Table 2:** Biomarkers in Functional Neurological Disorder: current evidence, potential clinical applications, and limitations.

Biomarker Domain	Type of Measure	Key Findings in FND	Potential Clinical Use	Limitations	Selected References
Neuroimaging	fMRI, resting-state connectivity	Altered connectivity between limbic, motor, and salience networks; abnormal activation of SMA and insula	Pathophysiological insight; subgroup identification	Not specific; group-level findings; limited reproducibility	[5,6,15,27]
Neuroimaging	Task-based fMRI	Abnormal emotion–motor coupling; altered self-agency processing	Mechanistic understanding of symptoms	Small sample sizes; heterogeneity of paradigms	[5,6,9,15,23]
Electrophysiology	EEG (PNES)	Absence of epileptiform activity during events	Differential diagnosis vs. epilepsy	Limited to seizure phenotype; not mechanistic	[14,15]
Autonomic markers	HRV, skin conductance	Altered autonomic regulation; increased arousal and stress responsivity	Monitoring physiological state; potential relapse markers	Low specificity; influenced by multiple factors	[10,28,29]
Neuroendocrine	Cortisol (HPA axis)	Dysregulated stress response, including hyper- or hypo-reactivity	Understanding stress-related vulnerability	Inconsistent findings; no diagnostic threshold	[25,31]
Behavioral/digital	Passive sensing, EMA	Changes in activity, social behavior, and sleep patterns	Monitoring symptom fluctuations; early detection models	Preliminary evidence; limited validation	[28,32]
Multimodal approaches	Combined neuroimaging + physiological + behavioral data	Emerging patterns integrating multiple systems	Toward personalized medicine	Exploratory; requires large-scale validation	[5,27,28,32]

Note: Most biomarkers remain at the research level and are not currently suitable for routine clinical diagnosis. Abbreviations. FND: Functional Neurological Disorder; fMRI: Functional Magnetic Resonance Imaging; SMA: Supplementary Motor Area; EEG: Electroencephalography; PNES: Psychogenic Non-Epileptic Seizures; HRV: Heart Rate Variability; HPA axis: Hypothalamic–Pituitary–Adrenal axis; EMA: Ecological Momentary Assessment.

## Data Availability

No new data were created.
